# Clinical implication of surgical resection for recurrent biliary tract cancer: Does it work or not?

**DOI:** 10.1002/ags3.12036

**Published:** 2017-09-14

**Authors:** Masaru Miyazaki, Hiroaki Shimizu, Hideyuki Yoshitomi, Atsushi Kato, Katsunori Furukawa, Tsukasa Takayashiki, Satoshi Kuboki, Shigetsugu Takano, Masayuki Ohtsuka

**Affiliations:** ^1^ Department of General Surgery Chiba University Chiba Japan; ^2^ Department of Gastroenterological Surgery Mita Hospital International University of Health & Welfare Tokyo Japan

**Keywords:** ampullary cancer, biliary tract cancer, cholangiocarcinoma, gallbladder cancer, recurrent cholangiocarcinoma

## Abstract

Although recent advances in imaging diagnosis, surgical techniques, and perioperative management can result in increased resectability and improved surgical outcomes, most resected patients still develop cancer recurrence. If patients develop cancer recurrence, their prognosis is very ominous. However, there have been some recent reports to show promising outcomes by aggressive surgical strategy in selected patients who developed cancer recurrence. Because there are various surgical procedures being selected at initial surgery in patients with biliary tract cancers, recurrent patterns after resection are very variable in each patient. However, surgical procedures might usually be very complicated and difficult if re‐surgical resection is considered in patients with recurrent biliary tract cancer, Therefore, surgical re‐resection could bring about high surgical morbidity and mortality rates in most previously reported series. Although re‐surgical resection might offer a chance of favorable outcome in selected patients with biliary tract cancers, these aggressive surgical approaches should be carried out in strictly selected patients by expert surgeons at high‐volume centers.

## 1 INTRODUCTION

Cholangiocarcinoma can be classified as intrahepatic cholangiocarcinoma, perihilar cholangiocarcinoma, or distal cholangiocarcinoma according to anatomical location. According to the previous Okuda classification, intrahepatic cholangiocarcinoma has been classified into peripheral and hilar types.^1^ Hilar type of intrahepatic cholangiocarcinoma is involved in the perihilar type of cholangiocarcinoma together with hilar cholangiocarcinoma. Peripheral intrahepatic cholangiocarcinoma (pICC) should be defined as intrahepatic cholangiocarcinoma (ICC) developed from peripheral intrahepatic bile ducts beyond the second‐order biliary radicle. Before 2001, pICC were coded as ICC and, subsequently, were coded as perihilar cholangiocarcinoma separately from ICC after implementation of the third edition of the International Classification of Diseases for Oncology.^2^ Therefore, the hilar type of ICC might be involved in perihilar cholangiocarcinoma together with hilar cholangiocarcinoma developed from the hilar extrahepatic bile duct. Extrahepatic bile duct could be defined as proximal bile ducts until the first‐order biliary radicle at the hilar region. Biliary tract cancer, including intrahepatic cholangiocarcinoma, gallbladder cancer, and ampullary cancer, is a devastating disease. Although recent advances in imaging diagnosis, surgical techniques, and perioperative management could bring about increased resectability and improved surgical outcome, most resected patients still develop cancer recurrence. If patients develop cancer recurrence, their prognosis is very ominous. However, there have been some recent reports to show promising outcomes by aggressive surgical strategy in some selected patients who developed cancer recurrence. Herein, these redo surgeries for recurrent biliary tract cancers are reviewed.

## 2 Intrahepatic Cholangiocarcinoma

The incidence of ICC has been increasing worldwide over the last several years.^3^, ^4^, ^5^, ^6^ Surgery is the only hope of achieving a cure in ICC. There are no survivors at 3 years in patients without surgical resection. Surgical resection has been shown to achieve a 3‐year survival of 40‐50%.^7^, ^8^ Tan et al.,^9^ using SEER Medicare data, identified 3756 patients with ICC, and only 12% underwent cancer‐directed surgery. In their study, 248 patients were identified with localized, potentially resectable disease (single, unilobar tumor without evidence of vascular invasion); however, only 91 (37%) of these patients underwent cancer‐directed surgery. Patients with localized disease who underwent cancer‐directed surgery had significantly improved median survival time (MST) (44 months vs 8 months; *P*<.01). As ICC is usually diagnosed at an advanced stage involving the hilar bile duct, hilar vasculature, hepatic vein, and inferior vena cava, hepatectomy combined with vascular and bile duct resection is usually required to obtain a negative surgical margin (R0).^10^ Major hepatectomy such as right, left hemihepatectomy or right or left trisectionectomy is also required for surgical resection of advanced ICC.^11^ Recently, a clinical trial of neoadjuvant chemotherapy has shown a downsizing or downstaging effect on initially unresectable locally advanced cholangiocarcinoma including ICC, and to make these tumors resectable. Kato et al.^12^ reported that gemcitabine chemotherapy was provided to 22 patients with initially unresectable locally advanced biliary tact cancer including ICC. Tumor was significantly downsized in nine patients, and surgical resection could be carried out in eight (36.4%) of 22 patients. MST in patients with surgical resection after downsizing chemotherapy and chemotherapy alone without surgical resection was 19.3 and 7.5 months, respectively (*P*<.05).^13^ Recently, major vascular resection has been feasible with acceptable surgical morbidity and mortality rates by expert hepatopancreatobiliary (HBP) surgeons at high‐volume centers such as combined resection of the inferior vena cava (IVC), hepatic vein, and the portal vein.^14^, ^15^ Combined IVC resection with hepatectomy could be safely done using appropriate vascular control methods such as total hepatic vascular exclusion, in‐vivo hepatic hyperthermic perfusion, and ex‐vivo combined IVC and/or hepatic vein resection with hepatectomy.^14^ Reconstruction of the IVC is repaired by ringed Goretex for segmental resection, and auto‐vein patch graft for partial resection. Retrohepatic IVC reconstruction after combined IVC resection below the confluence of the hepatic veins might not always be requisite as reported by Yoshidome et al.^16^ They reviewed 36 cases undergoing resection of the IVC concomitant with resection of malignancies. Among them, 10 patients underwent circumferential resection of the IVC. Most of the patients who did not undergo replacement of the IVC showed no sign of swelling of the lower limbs, but one patient showed persistent leg edema with oliguria. This patient had poor development of collateral circulation and mild obstruction of the IVC before surgery. They concluded that caval replacement after circumferential resection of the IVC may be necessary in patients who have preoperative development of collateral circulation or who have oliguria or unstable hemodynamics during surgery.

Whether surgical lymphadenectomy for ICC plays a beneficial role for survival in surgical resection is not clearly shown. Although patients with lymph nodal involvement had worse prognosis than those without lymph nodal involvement after surgical resection, surgical resection with lymphadenectomy in the patients with lymph nodal involvement was shown to be beneficial on the prognosis as compared with the prognosis of the unresectable patients.^11^ Therefore, lymph nodal involvement in patients with ICC might not be considered a good indication, but it might not be a contraindication to surgical resection. In the eighth edition of the AJCC staging system,^17^ regional lymph node N category is defined according to the hepatic location of ICC. For right‐liver ICC, regional lymph nodes include the hilar (common bile duct, hepatic arteries, portal vein, and cystic duct), periduodenal and peripancreatic lymph nodes. For left‐liver ICC, regional lymph nodes include infra‐phrenic, hilar and gastrohepatic lymph nodes. For ICC, spread to the celiac and/or periaortic and caval lymph nodes is distant metastasis (M1). In the seventh edition, gastrohepatic lymph nodes in left‐liver ICC is newly emerged as regional lymph nodes. This newly emerging definition was proposed according to the study of Tsuji et al.^18^

After surgical resection of ICC, high rates of recurrence of between 46% and 65% are reported.^19^ There are several prognostic factors reported for predicting survival after surgical resection: lymph node involvement, surgical margin, bile duct infiltration, intrahepatic metastasis, and CA19‐9. According to the study of Endo et al., the liver is the most common site of recurrence (63%).^20^ Other recurrent sites are intraperitoneal dissemination, commonly lymph nodes. Intrahepatic solitary recurrence without any other distant metastases might be a possible indication to repeat hepatectomy when future remnant liver volume after repeat hepatectomy is sufficient for a patient to withstand surgery. Some studies reported achieving a beneficial prognosis after repeat hepatectomy for solitary intrahepatic recurrence after initial hepatectomy for ICC.^21^ Surgical re‐resections have been reported in a small study series (Table 1). In these studies, recurrent site of ICC in most patients who underwent surgical re‐resection for the recurrent lesion was intrahepatic.^13^, ^19^, ^22^, ^23^, ^24^, ^25^, ^26^, ^27^, ^28^, ^29^, ^30^ Solitary or a few recurrent lesions in the liver only were surgically treated by repeat hepatectomy. Other recurrent sites have seldom been indicated for surgical resection. Several reports have shown that repeat hepatectomy for intrahepatic recurrence of ICC resulted in a beneficial effect on the prognosis. Spolverrato et al.^30^ reported a therapeutic result of 400 (71%) patients who developed recurrence among 563 patients undergoing curative‐intent hepatic resection for ICC. In their series, the first recurrence site was intrahepatic only (60%), extrahepatic only (15%), or intra‐ and extrahepatic (26%). Overall, 210 (53%) patients received best supportive care, whereas 190 (48%) patients received treatment such as systemic chemotherapy‐only (24%) or repeat liver‐directed therapy±systemic chemotherapy (76%). Median survival of patients undergoing resection of recurrent ICC was 26.7 months vs 9.6 months for patients who had intra‐arterial therapy. These results revealed that recurrence following resection of ICC is common, occurring in up to two‐thirds of patients. When there is recurrence, prognosis is poor. About 10% of patients had a possibility of repeat liver resection after recurrence, which brought about a modest survival benefit.

**Table 1:** Surgical re‐resection for recurrence of ICC after initial hepatectomy

Author	Year	n	Re‐surgery	Prognosis
Yamamoto et al.^13^	2001	4/25	Hepatectomy	17‐155 mo (range)
Ohtsuka et al.^22^	2009	9/37	Hepatectomy	Median 22 mo
Ercolani et al.^23^	2010	6/39	Hepatectomy	56% at 3 y
Kamphues et al.^24^	2010	13/71	Hepatectomy+Ablation	Median 51 mo
Saiura et al.^19^	2011	4/25	Hepatectomy	43% at 5 y
		1/25	Pneumonectomy	137 mo alive
Song et al.^25^	2011	5/74	Hepatectomy	6.8‐58.5 mo
Sulpice et al.^26^	2012	4/45	Hepatectomy	100% at 5 y
Takahashi et al.^27^	2015	2/47	Hepatectomy	NA
		3/47	Pneumonectomy	NA
		2/47	Locoregional	NA
Souche et al.^28^	2016	10/76	Hepatectomy	Median 25 mo
Miyazaki et al.^29^	2017	9/36	Hepatectomy	Median 28.5 mo (uncertain)
Spolverato et al.^30^	2016	41/400	Hepatectomy	Median 26.7 mo

ICC, intrahepatic cholangiocarcinoma; mo, months; n, no. patients for re‐surgery/recurrence; NA, not available; y, years.

Liver transplantation for ICC has been shown to result in very poor results by many studies. Five‐year survival after transplantation for ICC is approximately 30%.^31^, ^32^, ^33^ It is generally considered that the poor survival after liver transplantation for ICC does not justify the use of limited organ resources. A multicenter Canadian study by Ghali et al.^21^ investigated the outcome of incidental findings of ICC in primary sclerosing cholangitis explanted livers and reported a 3‐year survival of 30% and a median survival of 26 months. Currently, liver transplantation is not an established indication for ICC and should be considered only in the setting of a clinical trial. Of course, nowadays, liver transplantation should be considered a contraindication to recurrent ICC.

## 3 EXTRAHEPATIC BILE DUCT CANCER

Complete surgical resection, R0 resection of extrahepatic bile duct cancer is the most effective and only potentially curative treatment. With the advent of improved imaging diagnosis, and skilled surgical technique, surgical curative resection is more frequently applied and the surgical resection rate has increased gradually. However, the prognosis after surgical resection for bile duct cancer still remains unsatisfactory. Reported survival rates range from 24% to 40%.^34^, ^35^, ^36^ Recurrent biliary tract cancer usually manifests as local failure, liver metastasis, lymph nodes metastasis and peritoneal metastasis. Locoregional failure is the most common pattern of disease recurrence after margin‐negative resection. Liver metastasis is one of the common major causes of treatment failure in bile duct cancer, especially in the case of distal bile duct cancer and ampullary cancer.^37^ However, surgical results of hepatectomy for liver metastasis have not been fully justified. Kurosaki et al.^38^ reported 13 patients with bile duct cancer^7^ and ampullary cancer^6^ who underwent hepatectomy for hepatic recurrence after initial pancreaticoduodenectomy. In their series, four of the 13 cases survived more than 5 years. All four long‐term survivors underwent margin‐negative hepatectomy for a solitary metastasis and were given postoperative adjuvant chemotherapy. They concluded that hepatectomy for a solitary metastasis is the treatment of choice even after pancreaticoduodenectomy, but indication for hepatectomy for multiple metastases is still limited. In patients with extrahepatic bile duct cancer occupying the middle portion of the extrahepatic bile duct who underwent bile duct resection at initial surgery, pancreaticoduodenectomy or hepatectomy was carried out for recurrence of bile duct cancer in a small series including a case report. Takahashi et al.^27^ reported the results of surgery for recurrent biliary tract cancer on 34 patients with perihilar cholangiocarcinoma and 13 patients with distal bile duct cancer including seven intrahepatic cholangiocarcinomas and 20 gallbladder carcinomas. They showed that survival after recurrence was significantly better in the patients who had surgery than in patients without surgery (32% vs 3% at 3 years; *P<*.001). Survival after surgery for recurrence was similar between gallbladder cancer and cholangiocarcinoma; significantly better in patients with initial disease‐free interval >2 years; significantly worse in patients with chest or abdominal wall recurrence; and significantly better in patients with no lymph nodal disease in their primary cancer. Nodal status of the primary tumor and site of initial recurrence were identified as independent prognostic factors for recurrence after surgery. The authors advocated that surgical resection for recurrent biliary tract cancer can be carried out safely and offers a better chance of long‐term survival in selected patients (Table 2). Their series included all biliary tract cancer as intrahepatic cholangiocarcinoma, perihilar cholangiocarcinoma, distal cholangiocarcinoma, and gallbladder cancer that might be variously different in the biological behavior of malignancy and selected surgical procedures at initial surgery. Therefore, it might be more useful to consider the choice of appropriate therapy in a clinical case of biliary tract cancer recurrence by each of surgery for intrahepatic cholangiocarcinoma, perihilar bile duct cancer, distal bile duct cancer, and gallbladder cancer.

**Table 2:** Surgical re‐resection for recurrence of extrahepatic bile duct cancer after initial resection

Author	Year	n	Initial surgery	Re‐surgery	Prognosis
Targarona et al.^39^	1993	1/?	BDR	Hepatectomy	10 mo alive
Yoon et al.^40^	2005	1/?	BDR	Hepatectomy	46 mo alive
		1/?	Hepatectomy	BDR	9 mo alive
Hibi et al.^41^	2006	1	PD	Hepatectomy	8 mo alive
Hwang et al.^42^	2010	2/?	BDR	PD	37 and 65 mo alive
Song et al.^25^	2011	10/242	BDR	PD, LN dissection, 4‐101 mo (range)	
		10/242	BDR, PD	Metastasectomy	
Kureosaki et al.^38^	2011	7/37	PD	Hepatectomy	Median 14 mo
Noji et al.^43^	2015	18/114	NA	NA	23.5% at 5 y
Lee et al.^44^	2015	6/?	BDR	PD	Median 16 mo
Takahashi et al.^27^	2015	47/424	Hepatectomy, PD	PD, Hepatectomy	32% at 3 y
Miyazaki et al.^29^	2017	4/60	PD, hepatectomy	Hepatectomy	19% at 5 y

BDR, bile duct resection; HPD, hepatopancreaticoduodenectomy; LN, lymph nodes; mo, months; n, no. patients for re‐surgery/recurrence; NA, not available; PD, pancreaticoduodenectomy; y, years.

## 4 GALLBLADDER CANCER

Although the prognosis of patients with gallbladder cancer ranges widely and depends on disease stage, prognosis can be dismal even after surgical resection. The poor prognosis associated with gallbladder cancer is largely as a result of postoperative recurrence that can be as high as 30‐65% in certain patients.^45^, ^46^ Risk factors for predicting recurrence after surgical resection have been reported to be lymph node metastases, positive resection margin, moderate or poor tumor differentiation and a tumor located on the hepatic and bile duct sides. Margonis et al.^47^ reported rates and patterns of recurrence after curative intent resection for gallbladder cancer in a multi‐institutional analysis from the US Extrahepatic Biliary Malignancy Consortium. From their analysis, 76 patients (35%) experienced a recurrence: locoregional only 16%, distant only 66%, locoregional and distant 18%. Median time to recurrence was 9.5 months. On multivariate analysis, T3 disease, lymphovascular invasion and residual disease were associated with an increased risk of recurrence. Patients who recurred demonstrated a worse 1‐, 3‐, and 5‐year overall survival (OS) (1 year OS 91% vs 69%, 3‐year OS 79% vs 29%, and 5‐year OS 76% vs 16%). Receipt of adjuvant therapy was associated with improved overall survival among those patients who developed a tumor recurrence. The authors concluded that while chemotherapy did not decrease the rate of recurrence, patients who experienced recurrence after administration of adjuvant treatment faired better than patients who did not receive adjuvant therapy. Kim et al.^46^ also revealed risk factors influencing recurrence, and patterns of recurrence after radical resection in 166 patients for gallbladder carcinoma. In their series, regional lymph nodes and the liver were found to be the most common site for recurrence after curative resection. Lymph node metastases were identified as an independent predictor of tumor recurrence by multivariate analysis. Furthermore, in their retrospective study,^46^ it was shown that there was no significantly different disease‐free survival rate between the no‐adjuvant therapy group and the adjuvant therapy group. As to hepatic metastases from gallbladder cancer, Ohtsuka et al.^48^ showed by pathological study that the most important route in the development of hepatic metastasis from gallbladder cancer is along the portal tract after direct hepatic parenchymal invasion. Shirai et al.^49^ also reported that extent of microscopic angiolymphatic portal tract invasion correlates with gross depth of direct invasion of the liver. Therefore, the concept of “regional hepatic metastases”^50^, ^51^ might be considered to exist in the case of gallbladder cancer directly invading the liver parenchyma through the gallbladder bed. Surgical resection for this type of hepatic recurrence pattern after initial surgical resection for gallbladder cancer may be implicative for obtaining some beneficial effect on the prognosis. However, as yet, there is no promising evidence on the results of hepatectomy for hepatic metastases from gallbladder cancer. Very few reports are shown in the literature at the present time (Table 3).

**Table 3:** Surgical re‐resection for recurrence of gallbladder carcinoma after initial resection

Author	Year	n	Initial surgery	Re‐surgery	Prognosis
Noji et al.^43^	2015	9/36	NA	NA	23.5% at 5 y
Takahashi et al.^27^	2015	20/135	CHX‐HPD	NA	6% at 5 y
Miyazaki et al.^29^	2017	1/11	NA	NA	Median 28.5 mo

CHX, cholecystectomy; HPD, hepatopancreaticoduodenectomy; mo, months; n, no. patients for re‐surgery/recurrence; NA, not available; y, years.

## 5 AMPULLARY CANCER

Primary ampullary carcinomas are rare malignancies accounting for <1% of gastrointestinal cancers.^52^ Anatomically, ampullary cancer originates from Vater's ampulla, whereas periampullary tumors can originate from the bile duct, intestine or pancreas. Histologically, as the ampulla comprises two distinctive types of mucosa – intestinal and pancreaticobiliary – ampullary cancer originates from intestinal epithelium or pancreaticobiliary ductal epithelium. Ampullary cancer resection is generally feasible in 50‐60% of cases, compared to 10‐20% for pancreatic cancer.^53^, ^54^ Ampullary cancer has more favorable prognosis after surgical resection, with reported 5‐year overall survival rates of 37‐68% compared to only 10‐30% for resectable pancreatic cancer.^55^, ^56^ However, up to 50% of patients relapse after curative resection for ampullary cancer.^57^ Unfortunately, there are no standard options available in the postoperative management as a result of the rarity of the malignancy and the absence of prospective trials. Boone et al.^58^ showed a retrospective study to evaluate the role of reoperation in patients with recurrent periampullary cancer (Table 4). A total of 14 patients who had undergone prior resection were recruited and underwent palliative resection because of symptomatic recurrence. Postoperative morbidity was remarkably high. Complications (grade 3) occurred in eight patients and intraoperative mortality was 21.4%. Postoperative mortality reached 86% with median survival of 45 days. This study suggested that reoperation for palliative reasons is not recommended for these patients because of severe morbidity and high mortality rates. According to a study by Plastaras et al.^59^ reported that proton re‐irradiation seems to have a role in the management of locally recurrent ampullary cancer after resection and chemoradiation. However, their series included only 10 patients who underwent proton re‐irradiation for locally recurrent lesions.

**Table 4:** Surgical re‐resection for the recurrence of ampullary cancer after initial resection

Author	Year	n	Initial surgery	Re‐surgery	Prognosis
Kurosaki et al.^38^	2011	6/22	PD	Hepatectomy	8‐75 mo (range)
Miyazaki et al.^29^	2017	1/11	NA	NA	NA

mo, months; n, no. patients for re‐surgery/recurrence; NA, not available; PD, pancreaticoduodenectomy.

## 6 CONCLUSIONS

Biliary tract cancer usually recurs after surgical resection, even after curative resection. Because there are various surgical procedures being selected at initial surgery in patients with biliary tract cancers, recurrent patterns after resection are very variable in each patient. However, surgical procedures might usually be very complicated and difficult if re‐surgical resection is considered in patients with recurrent biliary tract cancer, Therefore, re‐surgical resection could cause high surgical morbidity and mortality rates in most previously reported series. Although re‐surgical resection might offer a chance of favorable outcome in some selected patients with biliary tract cancers, these aggressive surgical approaches should be carried out in strictly selected patients by expert surgeons in high‐volume centers.

## DISCLOSURE

Conflict of Interest: Authors declare no conflicts of interest for this article.
